# Supplementary material for: Biochemical characterization of an alkaline and detergent-stable Lipase from Fusarium annulatum Bugnicourt strain CBS associated with olive tree dieback
Source: PLoS One. 2023 May 19;18(5):e0286091. doi: 10.1371/journal.pone.0286091 (PMC10198573; doi:10.1371/journal.pone.0286091)
Supplement: S1 File — (PDF) [file pone.0286091.s001.pdf]

S1 Fig. SDS-PAGE raw image

1 2 3 4

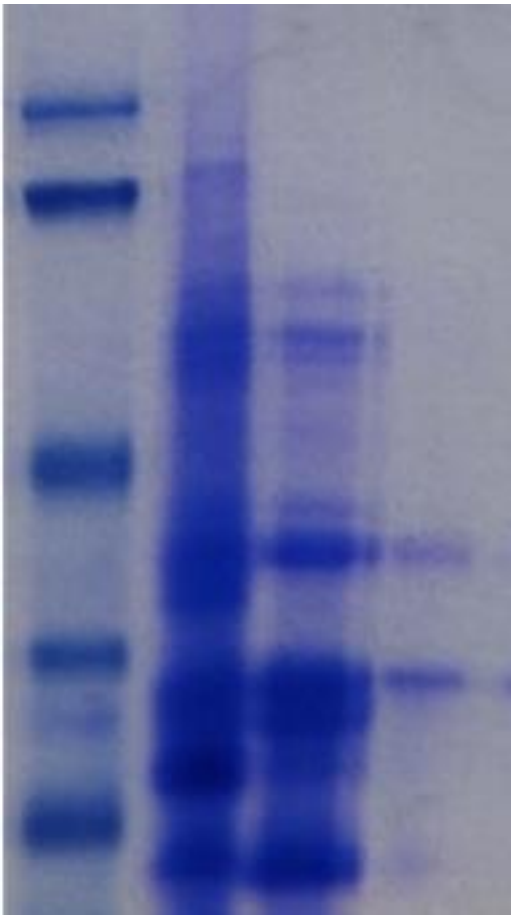

Loading order :

1. Molecular weight standard (#06P-0211, Euromedex)

2. Resuspended pellets after ammonium sulfate (80%)

3. Fraction obtained after gel filtration chromatography on Superdex® 200 Increase 10/300 GL column

4. Pooled active fractions from HiTrap™ Q-Sepharose FF column
