# Supplementary material for: Biochemical characterization of an alkaline and detergent-stable Lipase from Fusarium annulatum Bugnicourt strain CBS associated with olive tree dieback
Source: PLoS One. 2023 May 19;18(5):e0286091. doi: 10.1371/journal.pone.0286091 (PMC10198573; doi:10.1371/journal.pone.0286091)
Supplement: S2 File — (PDF) [file pone.0286091.s002.pdf]

S2 Fig. Zymography raw image

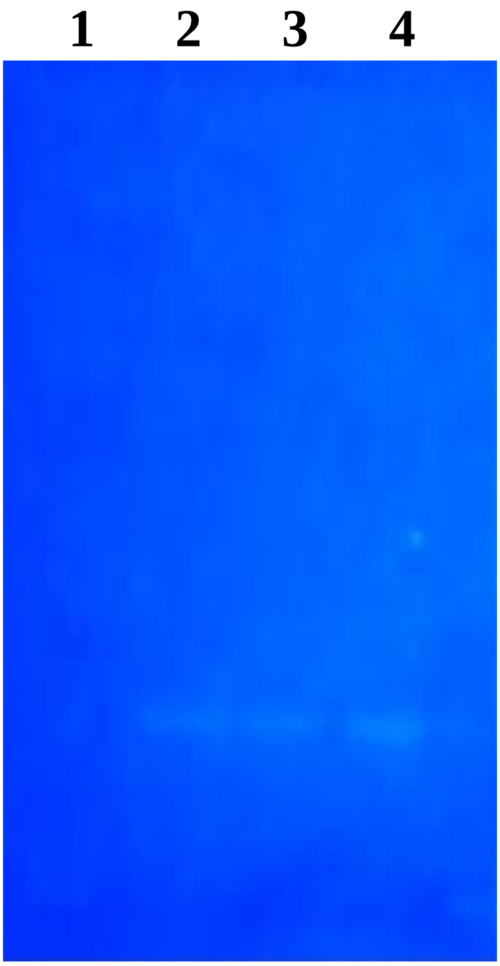

Loading order :

---

1. Control

2. Purified FAL active fraction no. 61 from HiTrap™ Q-Sepharose FF column

3. Purified FAL active fraction no. 62 from HiTrap™ Q-Sepharose FF column

4. Purified FAL active fraction no. 63 from HiTrap™ Q-Sepharose FF column
