# Supplementary material for: Biochemical characterization of an alkaline and detergent-stable Lipase from Fusarium annulatum Bugnicourt strain CBS associated with olive tree dieback
Source: PLoS One. 2023 May 19;18(5):e0286091. doi: 10.1371/journal.pone.0286091 (PMC10198573; doi:10.1371/journal.pone.0286091)
Supplement: Attachment — Submitted filename: Comments.docx [file pone.0286091.s003.docx]

**Comments**

Present study shows the production, purification and characterization of lipase from *Fusarium annulatum*. Since this study reports that purified enzyme had extreme tolerance to the presence of non-polar organic solvents, non-ionic and anionic surfactants and oxidants, in addition to significant compatibility and stability with some available laundry detergents; the present work is of great significance. This research paper can be accepted for publication in the Journal.

Few queries are as follows:

1. In section “Purification of FAL” my query is whether the mycelium was removed after centrifugation or before centrifugation? Filtration by using Whatmann filter Paper should be done prior to centrifugation.
2. In present study, Dialysis was not performed after ammonium sulfate precipitation. Why? Better results could have been obtained if the dialysis of protein sample was performed.
3. “*Fusarium*” and scientific names of other organisms should be italicized throughout the manuscript.
4. What is the advantage of isolating the lipase producing fungus from Olive oil trees over other sources? What motivated the authors to go for isolation from this particular source?
5. What was the purpose of extraction of olive oil/triolein in section “Washing Performance Analysis of FAL”?
